# Supplementary material for: Detecting the spin-polarization of edge states in graphene nanoribbons
Source: Nat Commun. 2023 Oct 21;14:6677. doi: 10.1038/s41467-023-42436-7 (PMC10590394; doi:10.1038/s41467-023-42436-7)
Supplement: Supplementary file 1 — Supplementary Information [file 41467_2023_42436_MOESM1_ESM.pdf]

Supplementary information for: Detecting the spin-polarization of edge states in graphene nanoribbons

Jens Brede,[1,2] Nestor Merino-Díez,[1,2] Alejandro Berdonces,[1,2] Sofía Sanz,[1] Amelia Domínguez-Celorrio,[3] Jorge Lobo-Checa,[3,4,5] Manuel Vilas-Varela,[6] Diego Peña,[6] Thomas Frederiksen,[1,7] Jose I. Pascual,[7,8,*] Dimas G. de Oteyza,[1,2,9,†] and David Serrate[3,4,5,‡]

[1]*Donostia International Physics Center, E-20018 San Sebastián, Spain*

[2]*Centro de Física de Materiales (MPC) CSIC-UPV/EHU, E-20018 San Sebastián, Spain*

[3]*Instituto de Nanociencia y Materiales de Aragón (INMA), CSIC-Universidad de Zaragoza,E-50009 Zaragoza, Spain*

[4]*Departamento de Física Materia Condensada, University of Zaragoza, E-50009 Zaragoza, Spain*

[5]*Laboratorio de Microscopias Avanzadas (LMA), Universidad de Zaragoza, E-50018, Zaragoza, Spain*

[6]*Centro Singular de Investigación en Química Biolóxica e Materiais Moleculares (CiQUS) and Departamento de Química Orgánica, Universidade de Santiago de Compostela; E-15782 Santiago de Compostela, Spain*

[7]*Ikerbasque, Basque Foundation for Science, E-48013 Bilbao, Spain*

[8]*CIC nanoGUNE BRTA, E-20018 San Sebastián, Spain*

[9]*Nanomaterials and Nanotechnology Research Center (CINN), CSIC-UNIOVI-PA, E-33940 El Entrego, Spain*

(Dated: October 5, 2023)

* ji.pascual@nanogune.eu

† d.g.oteyza@cinn.es

‡ serrate@unizar.es

CONTENTS

Supplementary Experimental Methods 2

Supplementary Theoretical Methods 7

Supplementary Note 1.- Edge states distribution in real and reciprocal space. 7

Supplementary Note 2.- Monitoring the magnetic state of tip and substrate. 12

Supplementary Note 3.- Electronic and magnetic interactions with the substrate 17

References 23

SUPPLEMENTARY EXPERIMENTAL METHODS

All measurements have been performed at the SPECS-JT-STM of the Laboratory for Advanced Microscopy (University of Zaragoza). The microscope, attached to the sample preparation chambers, features a base temperature of 1.17 K and an out-of-plane magnetic field up to 3 Tesla provided by a dry superconducting split-coil. The whole facility operates under ultra-high-vacuum conditions ($P \sim 1 \times 10^{-10}$ mbar). The tip is grounded and the tunneling bias V_b is applied to the sample. Data has been taken at $T = 1.2$ K unless stated otherwise. Differential tunneling conductance dI/dV is acquired using a lock-in amplifier at a frequency of 933 Hz and r.m.s. modulation given by V_{mod}. STM images and dI/dV maps were taken either in constant height or in constant current mode, as indicated at the corresponding caption for each data set. In the case of the constant current dI/dV maps as those shown in Fig. 2d of the main text and in Fig. S6b, the image is formed by slicing at fixed V_b the dI/dV signal from a dense grid where a full spectra is acquired ramping V_b at each pixel. All images have been analyzed using WSxM software package[1].

The Au(111) single crystal purchased from Mateck GmbH was cleaned by repeated Argon sputtering and annealing cycles at 510 °C. The $GdAu_2$ alloy is grown on the clean Au(111) surface by sublimating Gd using an e-beam source at a rate of approximately 0.06 ML/min while the substrate is held at 320 °C.

As shown in Fig. S1c, $GdAu_2$ forms a moiré superlattice caused by the superposition of its hexagonal unit cell (lattice parameter 5.41±0.03 Å along the $[\bar{2}11]_{Au}$ direction of the substrate) and the underlying Au(111) lattice[2, 3]. In the $GdAu_2$ lattice, each Gd atom is sixfold coordi-

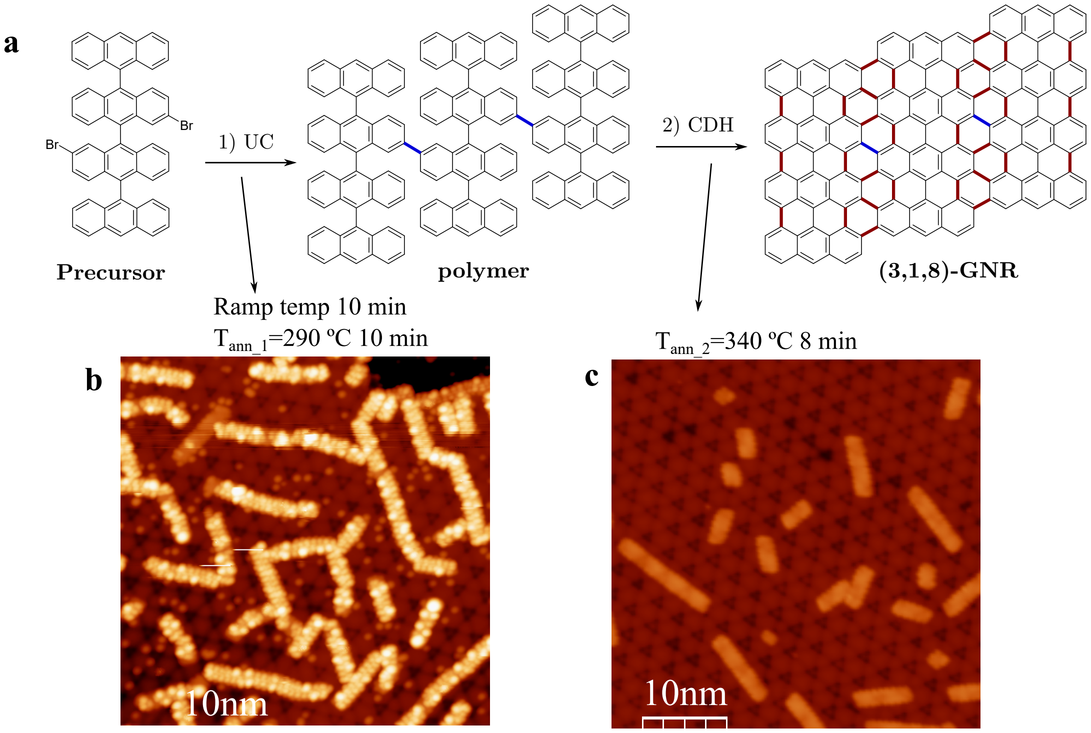

SUPPLEMENTARY FIGURE S1. (**a**) Reaction scheme of the surface catalyzed synthesis of (3,1,8)-chGNRs starting from deposition of the reactant 2”,3’-dibromo-9,9’:10’,9”:10”,9”’-quateranthracene (DBQA, see Fig. S1A), followed by Ullmann polymerization at approximately 290 °C, and subsequent cyclodehydrogenation (CDH) at 340 °C to achieve the final planar aromatic product. (**b**) Typical survey of the polymeric chains of DBQA (set point: -1.5 V, 100 pA). (**c**) Topography of fully planarized chGNR after CDH completion (set point: -1.5 V, 100 pA). (b) and (c) share the same color scale.

nated with Au atoms that are visualized as dark and bright spots respectively at $V_b = 1$ V (see Fig. S3a and Fig. 1b of the article). The moiré pattern can be approximated by 4 $GdAu_2$ unit cells in the $[1\bar{1}0]_{Au}$ direction on 13 Au(111) lattice constants, with a period of $d_m = 37.9 \pm 1$ Å, although our own atomically resolved images indicate that the moirée is not commensurate.

The reactant 2”,3’-dibromo-9,9’:10’,9”:10”,9”’-quateranthracene (DBQA) is then deposited on $GdAu_2$ from a home made resistive evaporator. Subsequent on-surface synthesis of (3,1,8)-chGNRs takes place in a two steps reaction as in the case of Au(111)[4], which is depicted in Supplementary Fig. S1.

Specifically, the temperature is slowly ramped up to 290 °C and then mantained for 10 minutes to obtain long polymers via Ullmann coupling. Fig. S1b shows the characteristic corrugated topography of these kind of polymers, surrounded by Br atoms coming from the dehalogenation of the DBQA that are still on the surface. Second, the inner hydrogens of the

polymer are cleaved by further heating up to 340 °C to form the C-C bonds marked in red, resulting in the final fully planarized chGNR shown in Fig. S1c. After cyclodehydrogenation we find (Fig. S2) 51 % of the GNRs aligned with their long edge parallel to high symmetry directions of the Gd atomic lattice (in the notation relative to the Au crystal, $[\bar{2}11]_{Au}$, as for example Fig. S6, and $[1\bar{1}0]_{Au}$, as for example Fig. 1B). Another 19 % are oriented at 13° from these directions (the case of the marked ribbon in Fig. 1c main text, or N=12 in Fig. S8), which corresponds to having the internal graphene lattice aligned with the high symmetry directions of the Gd mesh. The orientation of yet another ∼ 30 % of the GNRs could not be properly classified.

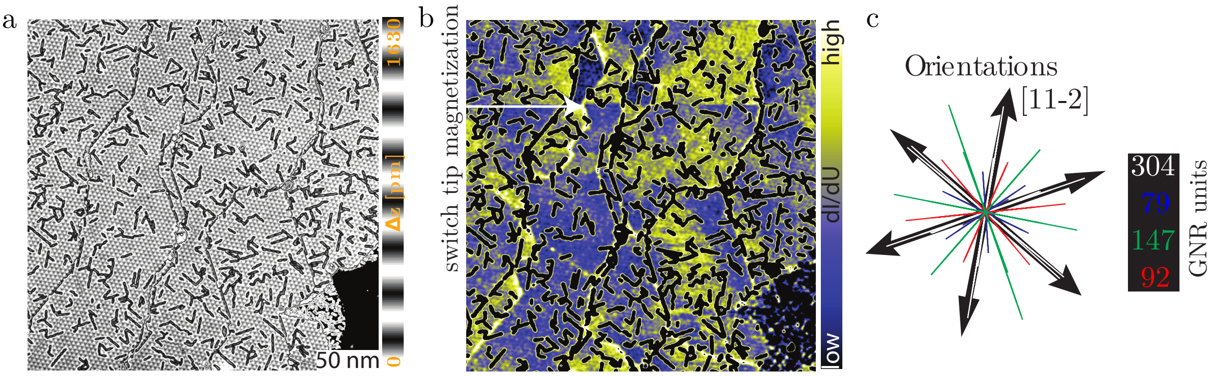

SUPPLEMENTARY FIGURE S2. Statistical study of the GNR orientation with respect to the $GdAu_2$ lattice. **a**) Large survey of (3,1,8)-chGNRs on several $GdAu_2$ terraces with B&W false color scale (V_b = 20 mV, I = 500 pA) **b**) Spin resolved dI/dV map of the same region (SP: 3 V, 50 pA; V_{mod} = 10 mV rms) showing the overall magnetic microstructure of the surface, taken with an in-plane sensitive Cr-tip. The white arrow indicates the line at which the tip's spin polarization changes sign. **c**) Polar representation of the orientation of the GNRs superimposed to the high symmetry crystallographic directions (black arrows). The length of the lines scales with the length (N) of the chGNRs observed in the various directions. The average length of chGNRs oriented along $[\bar{2}11]_{Au}$ (white) and $[\bar{1}10]_{Au}]$ (green) is about 30 % larger than for chGNRs that are 13° tilted with respect to them (blue and red). The total number of chGNRs analyzed is 891.

Spin polarized STM (SP-STM) has been carried out using bulk Cr tips. Tips are prepared by electrochemical etching of elongated pure Cr flakes and subsequent field emission cleaning (120 V, 1 μA, 1 hour) at the STM head.

$GdAu_2$ exhibit atomically sharp structural antiphase boundaries (APB) between crystallographic domains that are shifted by half a $GdAu_2$ lattice vector with respect to each other, as the one shown in Fig. S3a. In agreement with a previous SP-STM study of $GdAu_2$[5], we find that a vast majority of these APBs induce a local antiferromagnetic coupling of the neighboring

domains, as evidenced by the spin polarized map in Fig. S3b. We make use of this contrast to calibrate the spin sensitivity direction and spin polarization of the Cr tips, as described later in Supplementary Note 2. The tips are submitted to voltage pulses until the expected in-plane spin contrast of 20 % or more at $V_b = 3$ V shown in Fig. S3b is obtained.

dI/dV point spectra at equivalent locations of the domains with high (bright, B) and low (dark, D) differential conductance are presented in Fig. S3d. They provide the quantitatively spin resolved electronic structure in the range of -0.5 to 3.5 eV around the Fermi level. In Fig. S3e the spin asymmetry is calculated from the dI/dV spectra of representative moiré regions as $\%(B-D)/(B+D)$. The spin polarization at $V_b = 3$ V ranges 15 % in average, and changes to about -7 % at $V_b = 2.6$ V. This inversion of the spin polarization is also clearly seen in the constant height maps shown in Fig. S3c. Importantly, the spin polarization around the Fermi level is much smaller, of about 4 %. In addition, it is constant in a broad energy window of ± 0.25 eV and spatially homogeneous, as evidenced by the spin polarization map in Fig. S3c. This fact corroborates that the highly localized spin contrast and the spin polarization inversion discussed for chGNR's edge states in the main text are intrinsic to the ribbon, and not some kind of signal induced by the substrate. Note that, in addition, the ribbon plane probed by the tip in open feedback is $\sim$1.7 Å above the substrate, and therefore with a much smaller contribution than the carbon atoms themselves.

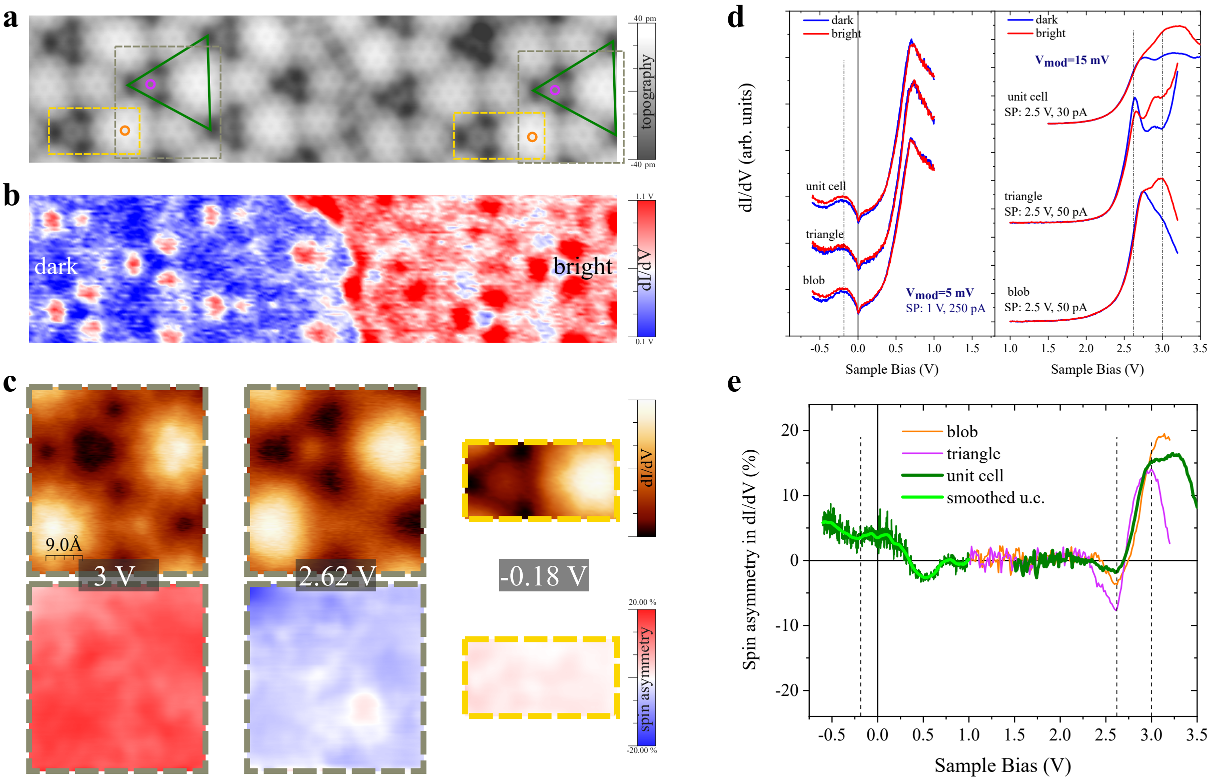

SUPPLEMENTARY FIGURE S3. (Study of the spin polarization of $GdAu_2$ (T = 1.2 K, in-plane sensitive bulk Cr-tip, B = 0 Tesla). **a**) Atomically resolved STM topography (SP: 1 V, 500 pA) of a region with a structural antiphase boundary (APB) separating two crystallographic domains that are shifted by half a $GdAu_2$ lattice vector. **b**) Constant current spin-resolved dI/dV map of the same region (SP: 3 V, 50 pA; V_{mod} = 20 mV rms) showing anti-parallel alignment of the magnetization across the APB. **c**) Constant height spin averaged (top row) and spin resolved (bottom row) dI/dV maps obtained from the dashed grey and yellow rectangles marked in the domains with darker (D, left side in A and B) and brighter (B, right side in A and B) spin polarized conductance. In all cases, the spin averaged DoS map is then obtained as $(B+D)/2$, whereas the spin asymmetry map is obtained as $\%(B-D)/(B+D)$. Stabilization set points before opening the feedback on top of the magenta circles in A are 1 V at 35 pA for the maps at V_b =3 and 2.62 V (V_{mod} = 20 mV rms), and 1 V at 1.5 nA for the map at $V_b = -0.18$ V (V_{mod} = 5 mV rms). Spin asymmetry color scale spans ±20 %. **d**) Low-bias (left panel) and high-bias (right panel) single point dI/dV spectroscopy obtained at the positions marked by the orange and magenta circles in A. The upper set of curves is an average over the approximate moiré unit cell depicted by the green triangles in A. Blue[red] curves correspond to the domains that are dark[bright] in B. Stabilization set points for the different spectra are given inside the graphs. **e**) Spin asymmetry calculated from the curves in D as $\%(B-D)/(B+D)$ for the following regions of the moiré pattern: center of the rounded blob (orange), center of the dark triangle (magenta) and approximate unite cell (green).

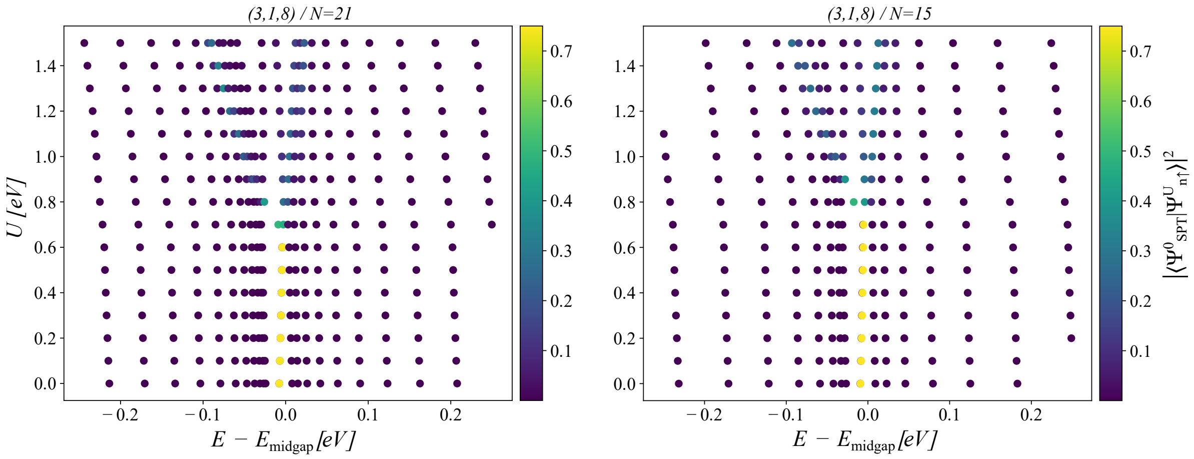

SUPPLEMENTARY FIGURE S4. Charged system (2 electrons added): Overlap of the eigenstates of the MFH Hamiltonian (obtained with different U) for $\sigma = \uparrow$ with the SPT state obtained with $U = 0$ (see eq. 1).

SUPPLEMENTARY THEORETICAL METHODS

As a consequence of electron doping, the original zero-energy topological end state (TES) state[4] of the neutral system is shifted to -8 meV after adding 2 electrons to the system (Fig. 2e main text). For increasing on-site Coulomb repulsion, the TES becomes hybridized with other orbitals at the following rate (color coded in Fig. S4, the yellow dot at $U = 0$ represents the pure TES)

$$\left|\langle \Psi^0_{\text{TES}} | \Psi^U_{n\uparrow} \rangle\right|^2 \tag{1}$$

where the Ψ^0_{TES} state corresponds to the unoccupied TES state and $\Psi^U_{n\uparrow}$ is the nth eigenstate for spin $\sigma = \uparrow$ –as defined in Eq. 3 of the main theoretical methods section– for varying U after addition of 2 electrons to the charge neutral ribbon. For both $N = 21$ and $N = 15$, Fig. S4 shows that the TES (yellow color) splits at a critical value of $U \sim 0.8$ eV and then mixes with other eigenstates above and below the Fermi level.

SUPPLEMENTARY NOTE 1.- EDGE STATES DISTRIBUTION IN REAL AND RECIPROCAL SPACE.

Constant height scans of the tunneling current portray a straightforward image of the density of states. However, in our case, this technique produces edge state images that are heavily

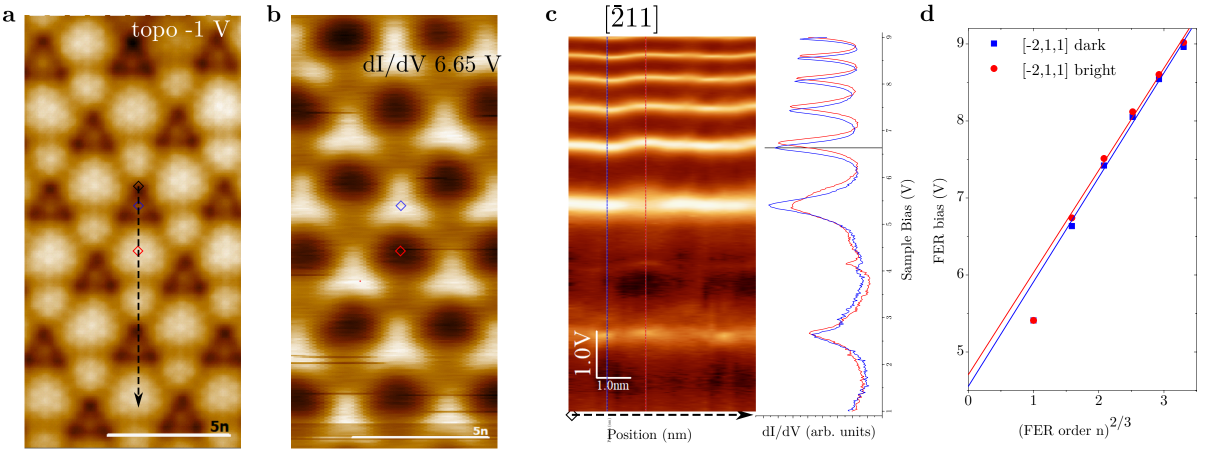

SUPPLEMENTARY FIGURE S5. (W-tip, temperature 4.3 K) **a** Atomically resolved STM topography of the $GdAu_2$ surface (SP=-1 V, 100 pA) **b** dI/dV map of the 2nd field emission resonance measured on top of the blue dot (SP: 6.65V, 2 nA, V_{mod} = 10 mV) **c** Stack plot of dI/dV spectra along the dashed line in (a), and selected point spectra at the blue and red positions (closed feedback, stabilization SP:1V, 500pA; V_{mod} =5 mV). The horizontal black line indicates the bias at which the map in (b) was taken. **d** Linear extrapolation of the FER bias to determine the local surface potential at the blue and red positions, following the model in Ref. 6.

influenced by the moiré pattern of the underlying $GdAu_2$ monolayer (see Fig. S6c). This is not related with the electronic structure of the edge states, but a consequence of the modulation of the local tunneling barrier induced by the moiré. To confirm this effect, we performed measurements of the local surface potential (ϕ_S) of the $GdAu_2$ by characterizing its field emission resonances (FER). Fig. S5 summarizes the correlation of ϕ_S with the moiré structure. A modulation of ∼50 meV can be observed in the 3rd to 6th FERs (Fig. S5c) between the bright parts and the dark triangles of the moiré. This shift is an approximation of the variation of ϕ_S. A better estimation can be retrieved from the back extrapolation to zero of the FER bias vs. the FER order to the power of 2/3[6], Fig. S5d, which yields $\phi_S = 4.71 \pm 0.14$ eV and $\phi_S = 4.55 \pm 0.15$ eV for brightest and darkest topographic moiré spots respectively. The full 2D correlation can be better appreciated in dI/dV maps tuning the bias to the exact energy of the 2nd FER at the position of the darkest part of the topography (the centre of any triangle in Fig. S5a), $V_b = 6.65$ V. In this way, Fig. S5b shows an inverted map of ϕ_S where the brightest regions correspond to a FER at exactly this energy, and the darkest regions are the same FER slightly shifted upwards in energy (and thus with higher ϕ_S), see dI/dV spectra in Fig. S5c.

The LDoS of the edge states of the chGNR will also be convoluted with the spatial variations of $\phi_S(x, y)$ discussed above when the data is taken in constant height mode. This effect can be partially suppressed working in constant current conditions, in which, for each pixel, the

tunneling resistance is kept constant by regulating at a set point of 150 mV and 125 pA before disabling the feedback and changing V_b to the value of interest. The resulting dI/dV signal as a function of V_b and longitudinal position is shown in Fig. S6b1. Figure S6 contains the analysis of the electronic structure in reciprocal space of the chGNR with $N = 21$ precursor units that is discussed in the main text. As shown in Fig. S6a, the edges (longitudinal axis) of this ribbon are parallel to the rows of Gd atoms (detected as bright spots in the dI/dV map at -100 mV, panel A) running along the $[\bar{2}11]$ direction of the substrate. We define the ribbon's physical edge as the line where the tunneling current (I_t) experiences a sharp drop (dull yellow and green lines) in constant height mode using a CO functionalized tip. In this particular case, the edge lies between two Gd rows, i.e., on top of Au atoms.

Line-wise Fast Fourier Transform (FFT) of the energy resolved LDoS of the edge (panel B.1) provides the 1D reciprocal space representation of the quasiparticle wave function modulus. In finite 1D ribbons, quantum confinement of the allowed k-vectors is expected to discretize the conduction and valence bands of the infinite counter parts, which have been reported elsewhere for (3,1,8)-chGNRs[4, 7]. For long enough ribbons, a collection of allowed k-vectors and their corresponding energies E, allows us to reconstruct the dispersion relation, and thereby the identification of the band which the peaks of interest in spectroscopy (Figs. 2c and 3d main text) come from. This study is summarized in Figs. S6b2-4. The $E(k)$ spots above 10 meV follow a parabolic dispersion with positive effective mass (pink guidelines in S6b). For $0 < E < 10$ meV, two additional spots (at k_1 and k_2) appear, which we ascribe to the moiré periodicity discussed earlier (see Figs. S6b2 and S6c). On top of them, a distinct intensity at $-10 < E < -6$ meV with $k \neq k_1$ is identified, which we associate to the onset of the conduction band because it matches the quadratic fit to the $E(k)$ dispersion at higher energies. The next experimental spots are found at E =-40 meV, and together with those at lower energies, fit well another quadratic $E(k)$ dispersion with negative effective mass. As a consequence, we ascribe the peaks at $E \leq -40$ meV to confined states belonging to the valence band.

This analysis justifies the assignment of the observed peaks in Fig. 2c of the main text to HOMO (-40 meV), LUMO/LUMO+1 (Fermi level) and LUMO+2 ($\sim$20 meV) of the charge neutral ribbon. The straightforward conclusion is that the chGNRs are slightly electron doped on $GdAu_2$/Au(111). The assignment is also supported by our LDoS simulations of the calculated edges states (Fig. S7). Owing to the sizable Coulomb repulsion ($U = 1$ eV, see Eq. 1 main methods section) required to describe precisely the DoS spectra (Fig. 2e main text), the confinement of edge states deviates from the trivial pattern of increasing integer number

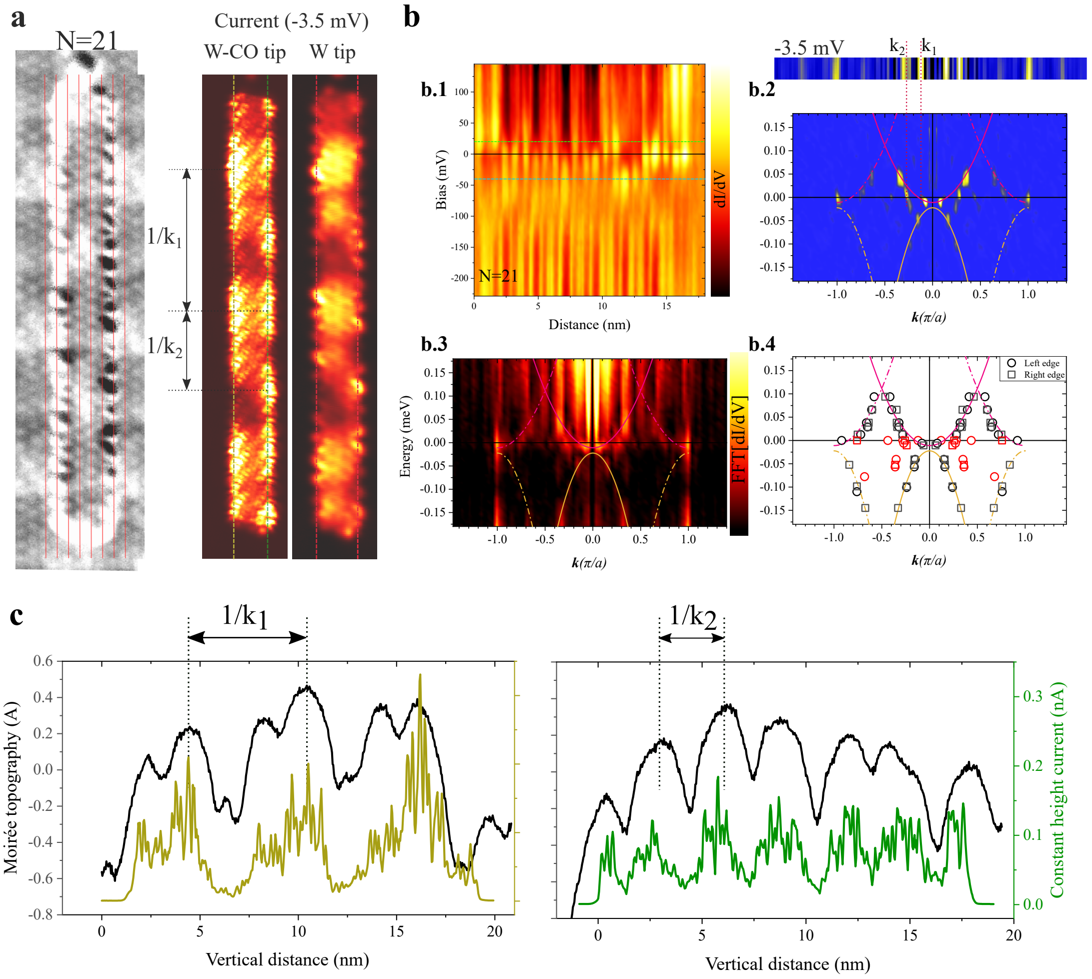

SUPPLEMENTARY FIGURE S6. **a** Left panel is a dI/dV *constant current* map of the $N = 21$ precursor units (3,1,8)-chGNR, oriented along $[\bar{2}11]_{Au}$ ($V_b = -450$ mV, current 500 pA, $V_{mod} = 10$ mV), where Gd atoms are visualized as bright circles (thin red lines mark Gd rows). Right panel shows the *constant height* tunneling current (I_t) of the same ribbon taken with a CO functionalized tip and a bare W tip (dull yellow and green dashed lines are the profile positions for panel (c), red dashed lines mark the line spectroscopy position utilized for the analysis in panel (b). **b** k-space analysis of the periodicities in dI/dV line spectroscopies taken along the red dashed lines in A (SP: 150 mmV, 125 pA, $V_{mod} = 10$ mV, $a = 8.87$ Å is the 1D lattice parameter of the ribbon). b1 shows a stack plot of dI/dV spectra as a function of the vertical distance in real space taken on the right edge. b2 is the line-wise fast Fourier transform (FFT) of b1 after background subtraction of the raw transformed image b3. b2 and b3 are average FFT of both edges. b4 is a collection of individual $E(k)$ spots obtained in the raw FT b3, and the pink and orange guidelines are best quadratic fits to the experimentally obtained $E(k)$. Red colored spots are excluded from the fit (see discussion in Supplementary Note 1). (b2) also includes a 1D FT of the edge I_t retrieved with the CO tip at -3.5 mV (average of both edges, profiles shown in panel (c), where the characteristic moiré pattern frequencies (k_1 and k_2) are indicated by red dotted lines. **c** Comparison of profiles of the moiré topography (SP: -500 mV, 500 pA) right below the ribbon's edges and of the constant height I_t at -3.5 mV taken with the CO-tip along the dashed lines in (a) of the same color. Notice the correspondence between the edge brightness and the characteristic repetition patterns of the moiré along these lines ($1/k_1$ and $1/k_2$, which are also identified in b2).

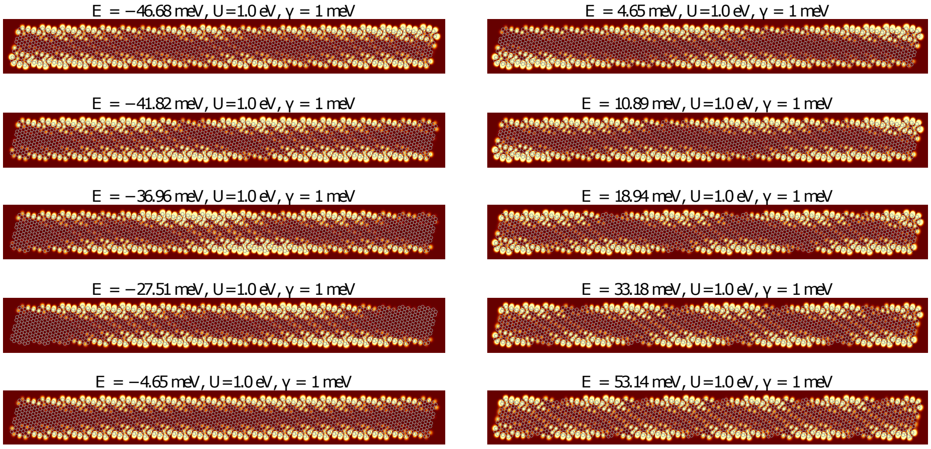

SUPPLEMENTARY FIGURE S7. LDoS summed over spin indices at the eigenstates (E_n, see from Eqs. 2 and 3 of the Main methods section) closest to the Fermi level for (3,1,8)-chGNRs of L=21 precursor units with a Coulomb repulsion parameter of $U = 1$ eV and an extra charge of $+2e^-$. The real space grid is sliced at $z =$ 3.5 Å above the molecular plane.

of nodal points for increasing energy in the proximity of the Fermi level. At higher (lower) eigenenergies, e.e. $E > 18$ meV ($E < -36$ meV), the usual sequence of an additional node per electron-like (hole-like) state is recovered.

The mixing of confined edge states as U increases in charged chGNRs is illustrated in Fig. S4 for $N = 21$ and $N = 15$. The non-interacting LUMOs heavily mix in the energy span of 20 meV around Fermi level. This effect is also reflected in the deviation of the experimental $E(k)$ from a free electron like quadratic dispersion, which is noticeable in Fig. S6b4.

Another remarkable observation is that metallic tips fail to produce a clear LDoS image of the chGNR eigenstates in constant height mode. When the I_t image is retrieved by metallic tips, it portrays sizable contributions from the substrate (Gd lattice is visible in the center) and the edge state is not evident, as opposed to the case of using CO-tips or constant current mode with metallic tips (see Figs. S6a and S8). The CO-tip, from its part, tunnels efficiently to the chGNR states, because it guarantees the small tip-sample distance required to resolve them in constant height mode[8]. For this latter case, the influence of the moiré pattern in the spatial distribution of the edge states discussed above is only negligible when the chGNR edge lies on top of Gd rows and parallel to the $[\bar{2}11]_{Au}$ direction ($N = 17$ in Fig. S8). On the contrary, for edges along $[\bar{2}11]_{Au}$ over Au rows (Fig. S6a), or tilted with respect to that direction ($N = 12$ in Fig. S8), the moiré pattern is replicated in the conductance profile.

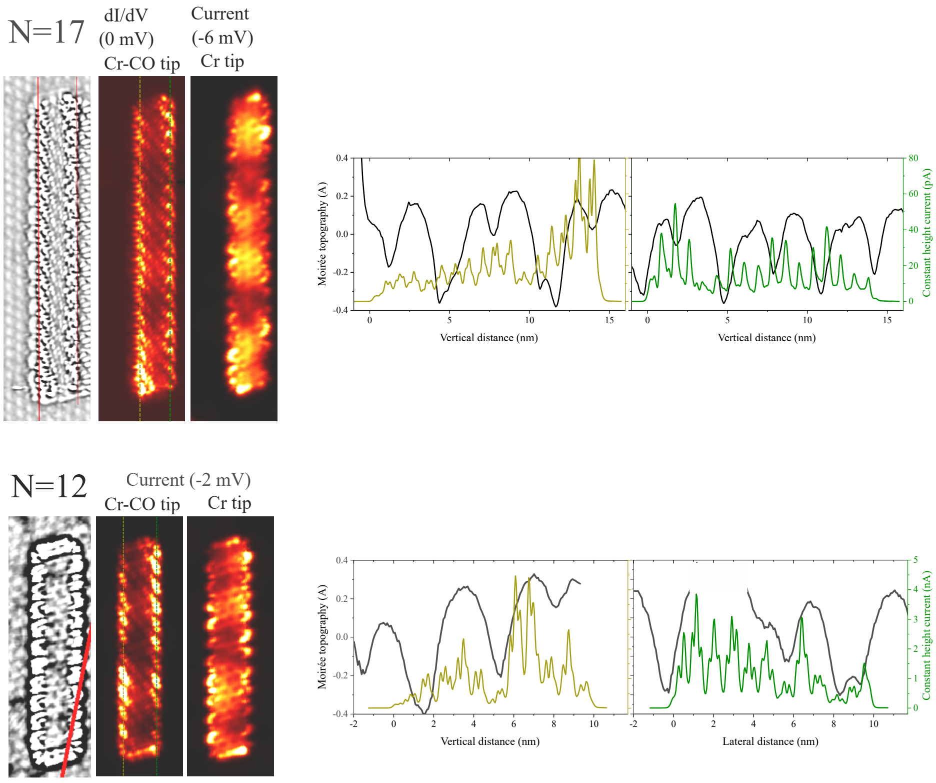

SUPPLEMENTARY FIGURE S8. Influence of the moiré pattern in constant height images of the $N = 17$ and $N = 12$ precursor units chGNRs with different stacking geometries over the substrate. In both cases, grey scale images are Laplace filtered STM topographies where the atomic Gd lattice and the ribbon perimeter can be visualized simultaneously (red lines mark Gd rows). The middle images are constant height DoS maps at Fermi level taken with a CO-functionalized tip Cr tip, while the right most images show the results for the bare metallic Cr tip. The profiles on the right panels display the moiré topography (SP: 20 mV, 500 pA) right below the ribbon's edges (black lines) and the constant height I_t at very low bias taken with the CO-tip along the dashed lines of the same color in the corresponding image.

SUPPLEMENTARY NOTE 2.- MONITORING THE MAGNETIC STATE OF TIP AND SUBSTRATE.

In order to measure the local spin polarization of the chiral edge states, the SP-STM characterization has been performed with exquisite control of the magnetic state of the tip and the supporting substrate. One example of the procedure is given in Fig. S9 concerning the $N = 15$ chGNR discussed in Fig. 3 of the main text. The characterization starts by taking a spin resolved image of the chGNR environment in the virgin magnetic state (prior to applying external magnetic field), as shown in panel A. Next, a ribbon characterization area (RCA) and a tip calibration area (TCA) are chosen. RCA has to be close enough to the ribbon, but not necessarily including it to avoid over-scanning the primary sample. The RCA (yellow box) con-

tains a control signal with the same spin dependent differential conductance as the $GdAu_2$ just beneath the chGNR. The TCA (green box) ought to contain one structural antiphase boundary (APB, see Fig. S3a,b) with antiferromagnetically coupled domains at either side. In this way, the TCA provides a well-defined contrast to track the tip sensitivity direction and its spin polarization, which is used to guarantee that the tip's spin is unchanged throughout the whole process, incluiding the field ramps.

We use the following notation (see sketch in Fig. S9b) for the magnetic state of the RCA (and therefore that of the ribbon's region): AP (or low dI/dV signal at $V_b = 3$ V, color coded blue) stands for the case in which the tip magnetiztation projection over the sample plane is mainly antiparallel to the RCA magnetization direction in the vicinity of the ribbon. P (or high dI/dV signal at $V_b = 3$ V, color coded red) stands for the parallel case. The different states of the same magnetic character are numbered according to the chronological order in which they were set up. All changes of magnetic state in RCA and TCA are achieved by driving the external field up to ± 2.8 T and then back to zero. Taking into account the strong easy-plane magnetic anisotropy of $GdAu_2$[9] with coercivity of the order of 20 mT[5], we attribute the field dependent contrast to the appearance of a small in-plane projection of the external field – caused by the unavoidable slight misalignement of the surface normal with respect to the vertical axis of the STM head –. To understand the evolution of the magnetic contrast in the TCA, it is divided in three magnetic domains (see Fig. S9c). Domain 1 (black dI/dV vs B curves), to the left of TCA, is separated from domain 2 (brown curves) by an atomically sharp APB, and it is always antiferromagnetically coupled to domain 1. Domain 3 (blue curves), to the right of TCA, is separated from domain 2 by a natural magnetic domain wall approximately 9 nm wide.

The evolution of the RCA magnetic state and the unaltered magnetization of the probe tip can be appreciated in full detail in Figs. S9b,c. The original virgin state of the RCA is $AP1$. Sharp jumps as a function of field in the dI/dV of the three domains correspond to local magnetization reversals caused by fast moving domain walls crossing the measurement area. Note that the magnetization reversals occurring in domain 1 and domain 2 are always simultaneous and of opposite sign. This is due to an antiferromagnetic exchange interaction across the APB. Alternatively, this local contrast evolution could be also caused by a spin flip of the tip apex. However, we can unambiguously rule out any change in the tip's probe spin because, when the reversal at the APB takes place, the magnetization of domain 3 remains constant (pinned by other distant APBs). The saturation contrast of all three domains (see

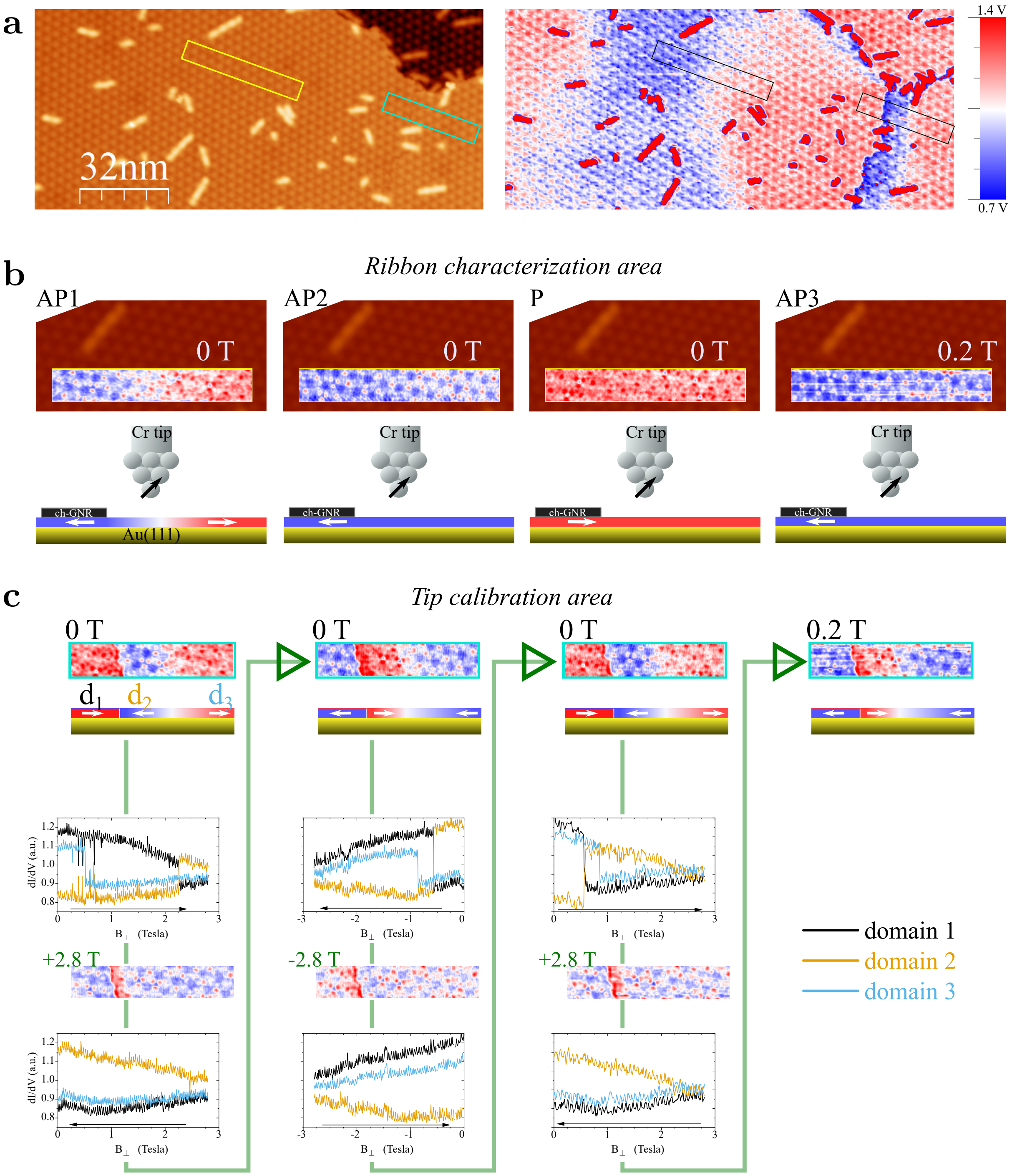

SUPPLEMENTARY FIGURE S9. Control of tip magnetic sensitivity during the acquisition of the data shown Fig. 3 main text ($V_b = 3$ V, 50 pA, $V_{mod} = 20$ mV, bulk Cr-tip). External field is perpendicular to the surface. The field variation history is indicated by the green arrows, and the field sweep direction by the black arrows in the graphs of panel (c). The box size for RCA and TCA are 44×7 nm^2 and 35×7 nm^2 respectively.

images at ± 2.8 T in panel c) is also illustrative in this respect. dI/dV in all domains experiences a slow monotonous evolution with increasing $|B|$ towards an intermediate value between those

of maximum contrast in remanence. This can be explained in terms of coherent rotation of the in-plane sample magnetization to become almost parallel to the field direction (while the tip sensitivity direction remains in-plane or slightly canted). This saturation takes place in fields above 2 Tesla, in good agreement with XMCD data of the Gd M_5 adsorption edge[9]. In all field sweeps, the depinning field of the domain wall between 3-2 is larger than the depinning field of the wall traveling through domain 1 ($\sim \pm 0.85$ T and $\sim \pm 0.5$ T respectively).

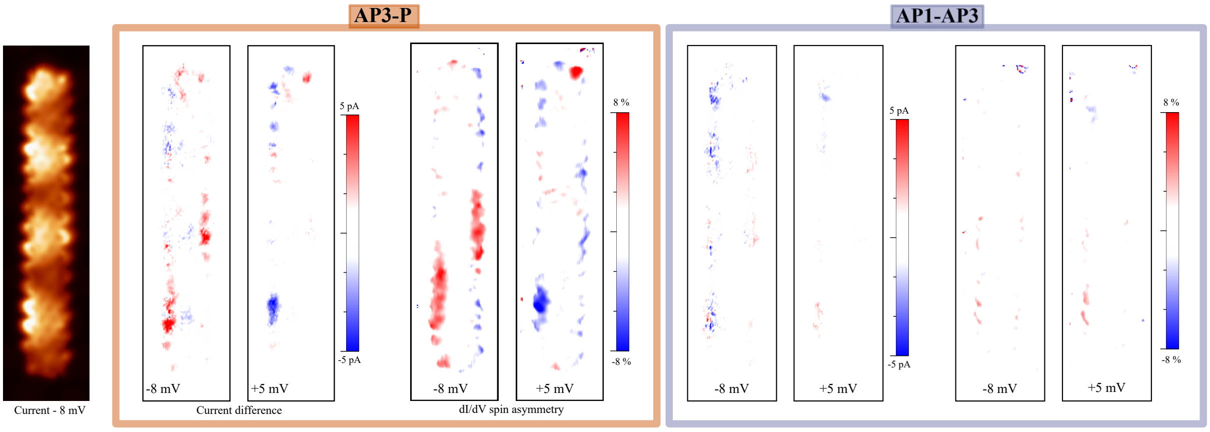

SUPPLEMENTARY FIGURE S10. Extended information about magnetic states in the experiment of Fig. 3, (in-plane sensitive bulk Cr-tip, $N = 15$ precursors). All images are 3.9×15.8 nm^2 and taken at constant height after opening the feedback over the ribbon's center (SP: 20 mV and 100 pA, $V_{mod} = 0.5$ mV). The panel $AP3 - P$ displays, at both sides of Fermi level, the magnetic contrast of the chGNR for two antiparallel magnetic states of the substrate. The direct current difference (left pair of images) carries the energy integrated spin polarization from Fermi level to the measuring energy, whereas the spin asymmetry (right pair of images) is proportional to the spin polarization right at the measuring energy. The panel $AP1 - AP3$ displays the direct current difference (left pair of images) and the spin asymmetry (right pair of images) for equivalent magnetic states of the substrate, resulting in the absence of spin contrast.

The first magnetization ramp exhibits a somewhat different behaviour than the successive ones: during the sweep $AP1 \rightarrow AP2$ the domain wall in RCA of the virgin state disappears, and the magnetization of the region between the ribbon an the TCA gets homogeneous, as evidenced in Fig. S9b. Afterwards, the magnetization of the RCA is homogeneous all the way to domain 1 and opposite to that of domain 2 in the TCA. In this way, the switch from $AP2 \rightarrow P$ is induced by sweeping the field down to -2.8 T and then back to zero, and the switch from $P \rightarrow AP3$ by the same procedure but up to positive field of +2.8 T.

In order to extract the magnetic contrast discussed in the main text (Fig. 3) we have used $AP3$ and P states. The result is reproduced in Fig. S10. Here, we plot the direct current difference $(AP3 - P)$ and the dI/dV spin asymmetry $(AP3 - P)/(AP3 + P)$. From this

procedure, only the magnetic contrast associated to the change $P \rightarrow AP3$ remains in the signal. Identical results are obtained when $AP1$ and P are compared. In contrast, when two images of equivalent magnetic states (like $AP3$ and $AP1$) are compared, there is no intensity left in either the current difference $AP3 - AP1$ or the dI/dV spin asymmetry $(AP3 - AP1)/(AP3 + AP1)$. This is unequivocal proof of the magnetic origin of the contrast observed in chGNR edges among in-equivalent magnetic states of the supporting $GdAu_2$.

SUPPLEMENTARY NOTE 3.- ELECTRONIC AND MAGNETIC INTERACTIONS WITH THE SUBSTRATE

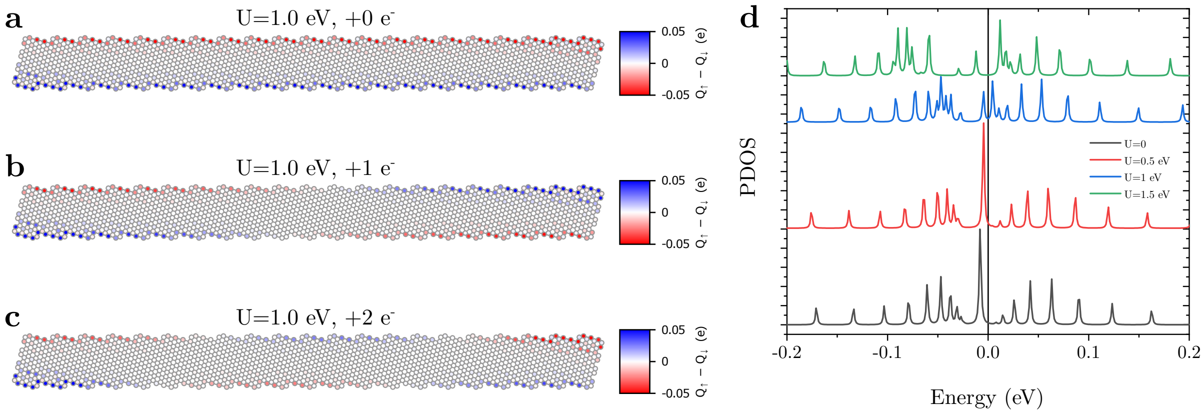

SUPPLEMENTARY FIGURE S11. **a,b,c** Spin polarization for (3,1,8)-chGNRs of $N = 21$ precursor units calculated with a Coulomb repulsion parameter of $U = 1$ eV. The different figures correspond to different charge states (neutral (a), +1e (b), +2e (c)), as indicated by the title of each panel. **d** Evolution of the site projected DoS (Eq. 2 main methods section) as a function of the U parameter for the case of a chGNR with two additional electrons and $N = 15$.

(3,1,8)-chGNRs belong to the class of graphene nanostructures with equal number of carbon atoms in both sublattices of the honeycomb structure. Therefore, Ovchinnikov's rule[10] and Lieb's theorem[11] impose that the total spin is zero, although the spin density may be locally finite. In particular, it is predicted the antiferromagnetic (AFM) alignment of the spin-polarized edge states on either side of the ribbon (Fig. S11a). However, one important ingredient of this theorem is the particle-hole symmetry (half-filling). In our case, we deviate from this situation in two aspects: first, the GNR is slightly electron doped, and the chemical potential lies above the charge neutrality point; second, the Hubbard Hamiltonian that reproduces the electronic structure shown in Fig. 2 includes hopping terms up to third nearest neighbours (see Eq. 1 in Theoretical Methods). Under these conditions, the magnetization of opposite edges do not necessarily add up to zero and Lieb's theorem does not hold[12–14]. Both deviations are small; the filling amounts to $2/21 \simeq 0.1$ electrons per unit cell (or ~ 0.03 electrons per zigzag lattice vector), while the ratio of second and third neighbor hopping relative to nearest neighbor one is ~ 0.07. However, it is enough to justify a finite but small spin-moment ascribed to the deviation from the expected AFM alignment between edges[13, 14]. Note that the long standing prediction of AFM coupling between edges was obtained in *ab-initio* and model Hamiltonian

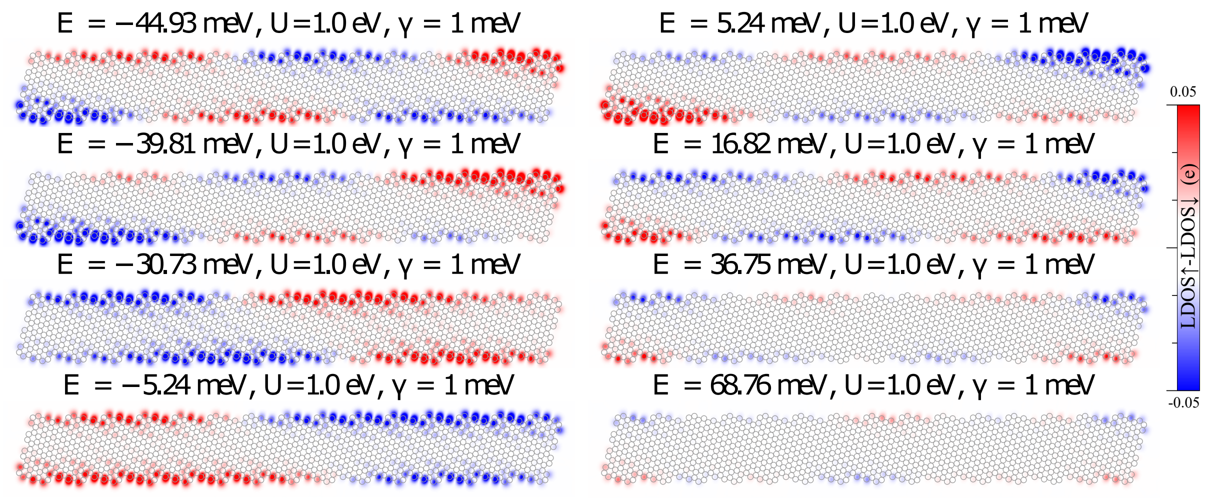

SUPPLEMENTARY FIGURE S12. Real space spin polarization at the eigenstates (E_n see Eqs. 2 to 4 of the main methods section, $\gamma = 1$ meV.) closest to the Fermi level for (3,1,8)-chGNRs of $N = 21$ precursor units calculated with a Coulomb repulsion parameter of $U = 1$ eV and an extra charge of $+2$ e^-. The real space grid is sliced at $z = 3.5$ Å above the molecular plane.

calculations only for the case of charge neutral ribbons. On the contrary, in zigzag GNRs with the same witdh as the (3,1,8)-chGNRs and small electron doping comparable to our case, MFH calculations find magnetic solutions where the magnetization of the edges are of opposite sign and uncompensated[14]. The finite spin moment of our electron doped chGNR could couple with the external magnetic field or undergo exchange interactions with the substrate. To rule out the influence of the external magnetic field we performed all measurements at remanence ($B_{ext} = 0$ T), exploiting opposite remanent states of the substrate (see Fig. 1).

There is another possible magnetic interaction: the exchange coupling with the substrate's magnetization. It may not only involve the total magnetic moment of the ribbon, but local variations of the interaction along the ribbon must also be taken into account. Specifically, there are two issues of interest related to our results. One is the lack of a structural registry between the graphene and the $GdAu_2$ lattices (noticeable in Figs. S6a and S8), which can produce spatial fluctuations of the exchange strength between carbon and $GdAu_2$ spin moments. The other one is the oscillatory edge magnetization caused by the the mixing of eigenstates. According to Fig. 2e, the charge neutral chGNR at half-filling has, to a very good approximation, particle-hole symmetry. The on-site Coulomb repulsion, parameterized here by U, preserves this symmetry, which means that in the absence of electron doping, the correlations splitting will increase symmetrically with respect to the Fermi level, avoiding the mixing of different quantum well states (with different number of nodal points) for moderate U values. In contrast, in our charged

model, higher order LUMOs start contributing to the near Fermi level peak as electrons are added to the chGNR (see Fig. S4), causing the total spin polarization to oscillate along the edge. This is shown in Supplementary Figs. S11a-c, where the number of sign changes of the spin polarization increases with the addition of electrons (Note that *e-e* correlations alone can not explain this behavior). The simulated spin polarization maps for each eigenstate displayed in Fig. S12) reproduce the inhomogeneities of the energy resolved spin-asymmetry images reported in Fig. 3e and Fig. S10. The experimental limit to the energy resolution imposed by the broad line-shape of the molecular states could explain the quantitative disagreement between theory and experiment in the energy resolved spin polarization.

To investigate the local exchange interaction between the chGNRs and their ferromagnetic support, we designed an experiment involving an intermediate state with in-homogeneous magnetization under the ribbon (i.e., the ribbon is crossing a natural domain wall of the $GdAu_2$), summarized in Fig. S13. Here, as shown in panel A for a $N = 22$ GNR, the magnetization of the $GdAu_2$ between P and AP states is opposite underneath the ribbon, but in the domain wall (DW) state the top part of the ribbon lies in a region with the same magnetization as the P state, whereas the bottom part lies in a region with the same magnetization as the AP state. In this way, we detect a small spin asymmetry $S_a > 0$ at Fermi level extracted from the difference ($P - DW$) only in the bottom part of the left edge (Figs S13c,d). For the ($AP - DW$) case, S_a is zero in the bottom part, and $S_a < 0$ in a small spot of the right edge at the top. Thereby, it can be concluded that the spin polarization does not behave like a strongly correlated spin density of the overall molecule, but it rather couples *locally* to the magnetic moments of the substrate.

More importantly, this interaction plays a crucial role to our work. It is responsible for stabilizing the magnetic moment of the edge states against thermal[15] and quantum fluctuations[16]. Available estimates of the spin-orbit coupling strength in graphene provide the figure of 15 μeV[17], which in a uniaxial approximation for the magnetic anisotropy would provide a energy barrier of the order of only 0.2 K. This means that the thermal fluctuations at any temperature near 0.2 K would suffice to destabilize spin moments in nanographenes[18]. In our experimental approach, however, this is not happening thanks to the exchange coupling to the $GdAu_2$. For ribbons positioned over homogeneous magnetization regions, as is the case for the experiment discussed in the main text, this magnetic interaction allows us to gain access to the edge's spin polarization. It provides a stationary magnetization which can be detected in slow SP-STM scans under different configurations of the tip-sample magnetization, and so, the demonstration

of magnetic remanence in the extended 1-D edge states of GNRs with high density of zig-zag segments. In order to get an experimental estimate of the energy scale for this interaction, we have carried out additional SP-STM experiments at a higher temperature of $T = 4.37$ K, displayed in Fig. S14. For a $N = 26$ (3,1,8)-chGNR, we obtained a sizable spin polarization with maximum value of $\mid S_a \mid \sim 5$ %. This suggests that the average exchange interaction between carbon atoms and the substrate must be significantly higher than the corresponding thermal energy of ∼0.4 meV, which leads to the conclusion that it determines the inter-edge spin alignment at a greater extent than small perturbations of the already weak internal AFM coupling.

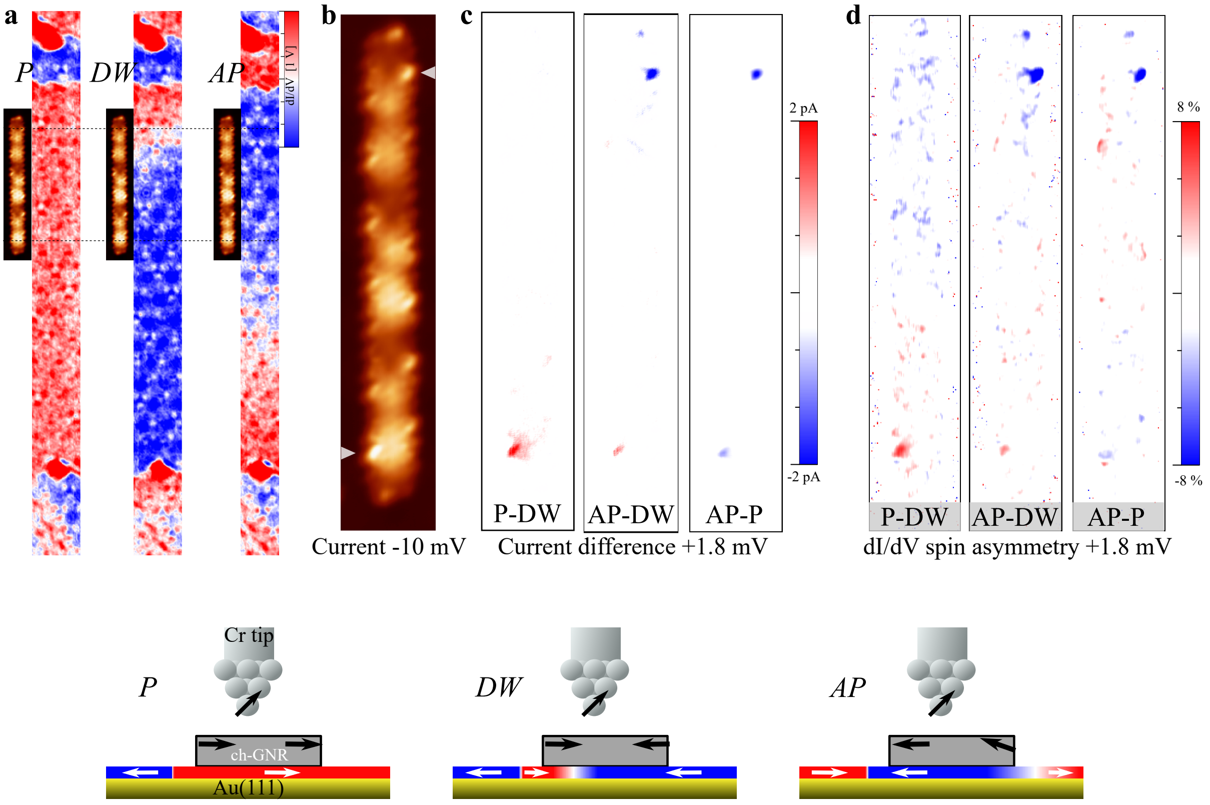

SUPPLEMENTARY FIGURE S13. **Magnetic interaction with the substrate.** Spin resolved DOS of a N=22 (3,1,8)-chGNR on GdAu2 (T=1.2 K, $B = 0$ T, bulk in-plane sensitive Cr tip) across a magnetic domain wall. **a** SP-STM dI/dV map (3V, 50 pA, $V_{mod} = 20$ mV, vertical length 80 nm) of the GdAu2 region immediately to the right of the ribbon (the small tunneling current image next to each map represents the true position of the ribbon) in three different magnetic states of the substrate obtained after different field cycles: P ($B = 0$ Tesla, minor cycle up to 2.3 T) exhibits in-plane magnetization parallel to the tip's magnetic moment in the region underneath the ribbon, AP ($B = 0$ Tesla, full cycle up to 3 T) shows mainly antiparallel alignment, while in the DW state ($B = 0$ T, full cycle up to -3 T) a natural domain wall about 10 nm wide has formed. **b** Tunneling current image of the N=22 (3,1,8)-cGNR at $V_b = -10$ mV. Triangles are placed at the same position as the dashed lines in (a) marking the spots where a finite spin asymmetry of the edge state has been detected. **c** Direct current difference at $V_b = 1.8$ mV between magnetic states P/AP and DW. **d** dI/dV spin asymmetry (S_a) calculated from the constant height differential conductance images in P,AP and DW states as $100 \times (P[AP] - DW)/(P[AP] + DW)$ at $V_b = 1.8$ mV ($V_{mod} = 0.5$ mV). All images in b,c,d (4×22 nm2) are taken constant height at the tip sample distance corresponding to a set point of 20 mV and 100 pA at the ribbon's center. In c,d positive S_a is observed in the bottom part of the ribbon only when comparing P and DW (see triangles in (b)), which is the position where P and DW states shows strong contrast in the $GdAu_2$ below the ribbon (see dashed lines in (a)). In the case of subtracting the images in AP state from the ones in DW state, we have the same result in the upper part of the ribbon, which is now the only region of the substrate with sizable magnetic contrast. For the $AP - P$ case, for which the whole ribbon is approximately over $GdAu_2$ with opposite magnetic moment, S_a in the top and bottom part behave as in the top part for $AP - DW$ case. In the spin averaged point spectra, these two spots are the only locations with strong intensity at Fermi level, suggesting that in the rest of the edge no traces of spin polarization are expected.

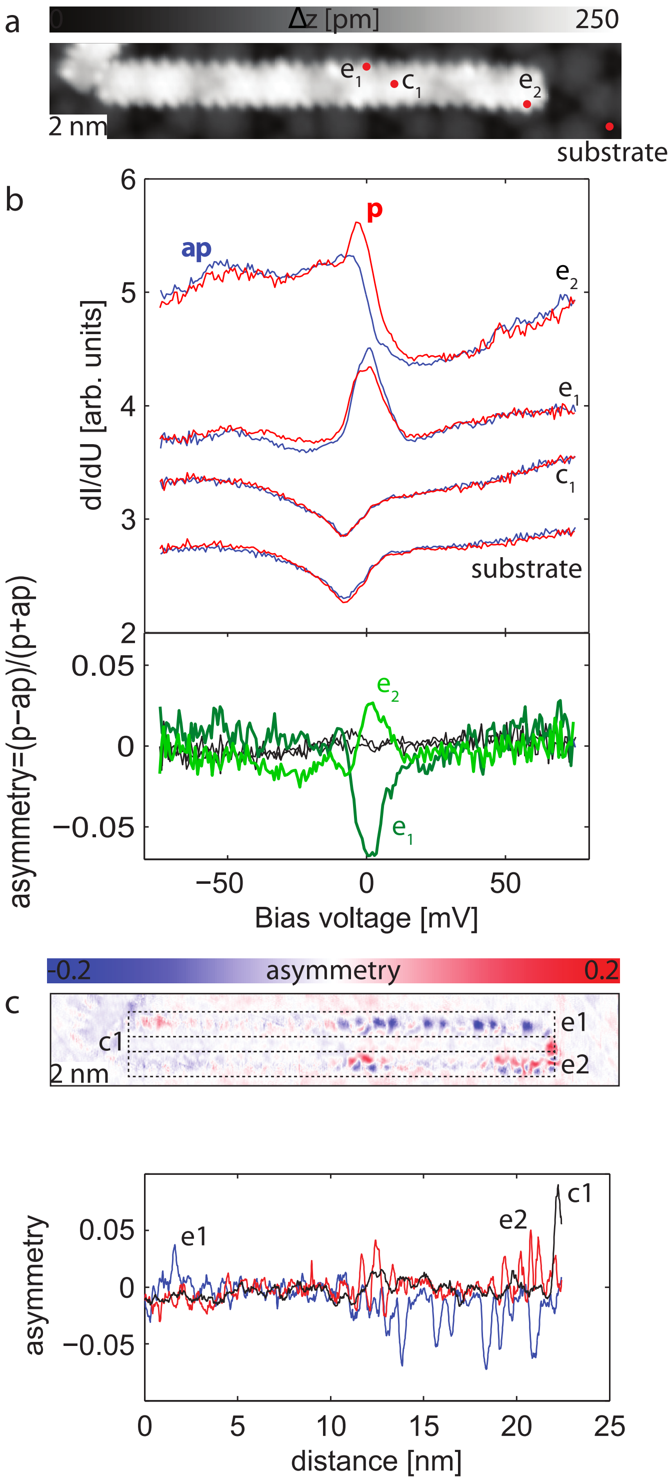

SUPPLEMENTARY FIGURE S14. **Spin polarization at 4.3 K.** (Cr-tip) **a** Topography of a (3,1,8)-chGNR with 26 PU units length on $GdAu_2$ (SP: 75 mV, 300 pA). **b** High resolution dI/dV spectra at the positions marked in (a) for opposite magnetic states of the substrate P and AP (top panel), together with the calculated spin asymmetry as $(P - AP)/(P + AP)$(bottom panel) (stabilization SP: 20mV, 500 pA; $V_{mod} = 1$ mV rms). **c** Constant height maps of the spin asymmetry calculated from the dI/dV images in P and AP magnetic states of the tip-sample system, $V_b = 2.5$ mV ($V_{mod} = 1$ mV rms).

[1] I. Horcas, R. Fernández, J. M. Gómez-Rodríguez, J. Colchero, J. Gómez-Herrero, and A. M. Baro, Wsxm: A software for scanning probe microscopy and a tool for nanotechnology, Review of Scientific Instruments **78**, 013705 (2007).

[2] M. Corso, M. J. Verstraete, F. Schiller, M. Ormaza, L. Fernández, T. Greber, M. Torrent, A. Rubio, and J. E. Ortega, Rare-earth surface alloying: A new phase for $gdau_2$, Phys. Rev. Lett. **105**, 016101 (2010).

[3] M. Corso, L. Fernández, F. Schiller, and J. E. Ortega, Au(111)-based nanotemplates by gd alloying, ACS Nano **4**, 1603 (2010).

[4] J. Li, S. Sanz, N. Merino-Díez, M. Vilas-Varela, A. Garcia-Lekue, M. Corso, D. G. de Oteyza, T. Frederiksen, D. Peña, and J. I. Pascual, Topological phase transition in chiral graphene nanoribbons: from edge bands to end states, Nature Commun. **12**, 5538 (2021).

[5] M. Bazarnik, M. Abadia, J. Brede, M. Hermanowicz, E. Sierda, M. Elsebach, T. Hänke, and R. Wiesendanger, Atomically resolved magnetic structure of a gd-au surface alloy, Phys. Rev. B **99**, 174419 (2019).

[6] T. König, G. H. Simon, H.-P. Rust, and M. Heyde, Work function measurements of thin oxide films on metals—mgo on ag(001), The Journal of Physical Chemistry C **113**, 11301 (2009).

[7] O. V. Yazyev, R. B. Capaz, and S. G. Louie, Theory of magnetic edge states in chiral graphene nanoribbons, Phys. Rev. B **84**, 10.1103/PhysRevB.84.115406 (2011).

[8] H. Söde, L. Talirz, O. Gröning, C. A. Pignedoli, R. Berger, X. Feng, K. Müllen, R. Fasel, and P. Ruffieux, Electronic band dispersion of graphene nanoribbons via fourier-transformed scanning tunneling spectroscopy, Phys. Rev. B **91**, 045429 (2015).

[9] L. Fernández, M. Blanco-Rey, M. Ilyn, L. Vitali, A. Magaña, A. Correa, P. Ohresser, J. E. Ortega, A. Ayuela, and F. Schiller, Co nanodot arrays grown on a gdau2 template: Substrate/nanodot antiferromagnetic coupling, Nano Letters **14**, 2977 (2014).

[10] A. A. Ovchinnikov, Multiplicity of the ground state of large alternant organic molecules with conjugated bonds, Theoretica Chimica Acta **47**, 297 (1978).

[11] E. H. Lieb, Two theorems on the hubbard model, Phys. Rev. Lett. **62**, 1201 (1989).

[12] A. R. Carvalho, J. H. Warnes, and C. H. Lewenkopf, Edge magnetization and local density of states in chiral graphene nanoribbons, Phys. Rev. B **89**, 245444 (2014).

[13] K. Sawada, F. Ishii, M. Saito, S. Okada, and T. Kawai, enPhase Control of Graphene Nanoribbon by Carrier Doping: Appearance of Noncollinear Magnetism, Nano Letters **9**, 269 (2009).

[14] J. Jung and A. H. MacDonald, enCarrier density and magnetism in graphene zigzag nanoribbons, Phys. Rev. B **79**, 235433 (2009).

[15] F. Meier, L. Zhou, J. Wiebe, and R. Wiesendanger, Revealing magnetic interactions from single-atom magnetization curves, Science **320**, 82 (2008).

[16] F. Donati, S. Rusponi, S. Stepanow, C. Wäckerlin, A. Singha, L. Persichetti, R. Baltic, K. Diller, F. Patthey, E. Fernandes, J. Dreiser, Ž. Šljivančanin, K. Kummer, C. Nistor, P. Gambardella, and H. Brune, Magnetic remanence in single atoms, Science **352**, 318 (2016).

[17] M. Slota, A. Keerthi, W. K. Myers, E. Tretyakov, M. Baumgarten, A. Ardavan, H. Sadeghi, C. J. Lambert, A. Narita, K. Müllen, and L. Bogani, Magnetic edge states and coherent manipulation of graphene nanoribbons, Nature **557**, 691 (2018).

[18] O. V. Yazyev and M. I. Katsnelson, Magnetic correlations at graphene edges: Basis for novel spintronics devices, Phys. Rev. Lett. **100**, 047209 (2008).
